# Film Distillation with a Porous Condenser for Seawater Desalination: Evaluation of Materials’ Stability in the Tropical Climate of Vietnam

**DOI:** 10.3390/membranes13020163

**Published:** 2023-01-27

**Authors:** Denis Kalmykov, Tatyana Anokhina, Ilya Borisov, Hoang Thanh Long, Trong Dan Nguyen, Alexey Volkov

**Affiliations:** 1 A.V. Topchiev Institute of Petrochemical Synthesis, Russian Academy of Sciences, Leninsky pr. 29, 119991 Moscow, Russia; 2 Joint Russian–Vietnamese Tropical Research and Technology Center, South Branch, Ho Chi Minh City 740500, Vietnam

**Keywords:** desalination, membrane methods, seawater preparation

## Abstract

Desalination and treatment of wastewater has become critical for Asia regions with water scarcity. In this work, the concept of thin-film distillation equipped with a porous condenser (FDPC) was considered for its implementation in a tropical climate of Vietnam. It was found that samples with a concentration of biocide of 0.5 wt.% possessed lower biofouling, in contrast to the neat membranes. The FD-PC module was developed and water desalination experiments were conducted in Russia and Vietnam. The experiments showed high reproducibility of the results; in particular, the evaporation rate was (4.9/3.0) kg/m^2^h in Russia and (4.1/2.0) kg/m^2^h in Vietnam. In addition, as part of this work, the optimal configuration of the installation was calculated using solar collectors as the main energy source. The calculation showed high energy efficiency: specific energy consumption 0.1–0.5 kWh/m^3^.

## 1. Introduction

The growth of the world’s population, increased sea level, intense use of fertilizers and chemicals in agriculture, and industrial and urban wastes lead to a shortage of high-quality freshwater and an increase in soil salinity. This is becoming a critical problem for several Asian, African, and MENA countries [1]. In particular, up to 57% of farms in Vietnam face limited access to fresh water and suffer from increased salinity of fertile soil [2,3]. At the same time, the groundwater in Vietnam, especially near agricultural lands, is significantly contaminated with herbicides, arsenic compounds, and other toxic substances [4]. Thus, the desalination and treatment of wastewater has become critical for regions with water scarcity such as Vietnam. To address this need, the membranes are effectively used globally to produce about 95 million m^3^ of fresh water per day from seawater by reverse osmosis [5]. 

Unfortunately, reverse osmosis (RO) has a number of disadvantages, especially for application in remote areas with a shortage of electricity, and requires seawater pretreatment, elevated pressures, and utilization of brine solution [6,7,8,9,10]. Bearing this in mind, the membrane-assisted distillation process can be considered as a potential alternative to RO, which does not require high-pressure operation and can utilize waste heat or solar radiation [11,12,13,14,15]. However, membrane distillation can be successfully operated once the membrane pores are not wetted by the water, which makes it difficult to maintain it during long-term operation because of fouling issues. To overcome this problem, a novel approach was recently proposed that combines thin-film distillation equipped with a porous condenser (FD-PC) [16]. In this configuration, the water is evaporated from the surface of a thin liquid film supplied on the heating plate, while the water vapors are condensed on the cold porous surface situated at a distance of a few millimeters, allowing to evacuate the evaporated water from the system.

As the feed solution does not come into contact with the membrane, and evaporation takes place from the whole surface of the liquid phase, FD-PC configuration does not possess the disadvantages typical of membrane distillation, such as wetting of the pores or the latent heat loss via membrane material. The porous membrane is used for condensation and evacuation of condensed water vapors, thus it is not exposed to the salt solution. Therefore, this implementation of the process makes it possible to achieve high process efficiency regardless of the complexity of the composition of saline water. A thin film distillation system can be used for the treatment of high salinity and complex mixtures by using low-pressure circulation pumps [11,17]. Besides, this system is flexible in operation and does not require a high constant temperature; therefore, the solar collectors with a sufficiently high efficiency can be used as a heating element in the case of a relatively small difference between the heated solution and the air temperature. The results of previous studies [18] devoted to film distillation have shown the high efficiency of this method. Therefore, permeate flux was observed from 4.9 to 6.2 kg/m^2^h at a solution heating temperature of 60. Bearing this in mind, the FD-PC seems to be a promising approach for application in remote areas [19] by utilizing renewable sources of energy such as wind power and solar radiation.

The photothermal membrane distillation (SVGMD) process also has similar properties. This method is of considerable interest from the point of view of energy consumption. Therefore, according to the data presented in [20], they show performance indicators comparable to FD (about 3.8 kg/m^2^h).

Within this work, the applicability of the innovative method of film distillation with a porous condenser for the process of desalination of water in the tropical climate of Vietnam is investigated. This method is of great interest from the point of view of low specific energy consumption and, therefore, the method is interesting from the point of view of using renewable energy sources. As goals of this work, the stability of structural materials was studied, and the module performance was analysed directly in a tropical climate.

It is important to take into account that biofouling is a critical problem in the membrane treatment of seawater and wastewater as it significantly reduces the efficiency of purification processes [21]. The production and application of polymer composite materials with biocide properties are of considerable practical interest both to ensure the resistance of construction materials to biological corrosion and to improve sanitary and epidemiological control. Some polyelectrolytes, particularly polyguanidine, are biocide active compounds; besides, compounds based on guanidine monomers are characterized by high biocide efficiency but, at the same time, low toxicity. Besides, the membranes containing guanidine as a biocide additive are drawing increasing attention nowadays [22]. The goal of this work was to develop component materials and improve the thermogradient method of desalination of seawater in a tropical climate using materials resistant to biocorrosion and biofouling by microorganisms.

## 2. Materials and Methods

### 2.1. Climatic Conditions of Vietnam

Environmental stability tests of samples in tropical conditions of Vietnam were carried out at three climatic testing stations (CTS):

- Dam Bai (Nha Trang)—south of the country, marine coastal climate;
- Hoa Lak (Hanoi)—north, humid tropical climate;
- Con Zo (Ho Chi Minh city)—south of the country, mangrove forests.

The tests were carried out by exposing the samples in the following 4 modes:

(1) outdoors (hereinafter referred to as “sun”);
(2) outdoors under a canopy (hereinafter referred to as “shade”);
(3) submerged in seawater to a depth of ~1 m (hereinafter referred to as the “sea”);
(4) in the ground (hereinafter referred to as “ground”);

The exposure time varied; the control of samples was carried out in stages of 5, 8, and 13 months.

### 2.2. Materials

Polyethylene terephthalate glycol (Z-PETG, d = 1.75 mm) was made by Zortrax SA, Olsztyn, Poland. 

Polysulfone, polyethersulfone, and polyphenylene sulfone were provided by BASF (Ludwigshafen, Germany).

### 2.3. Thin Film Distillation

**Figure 1:**
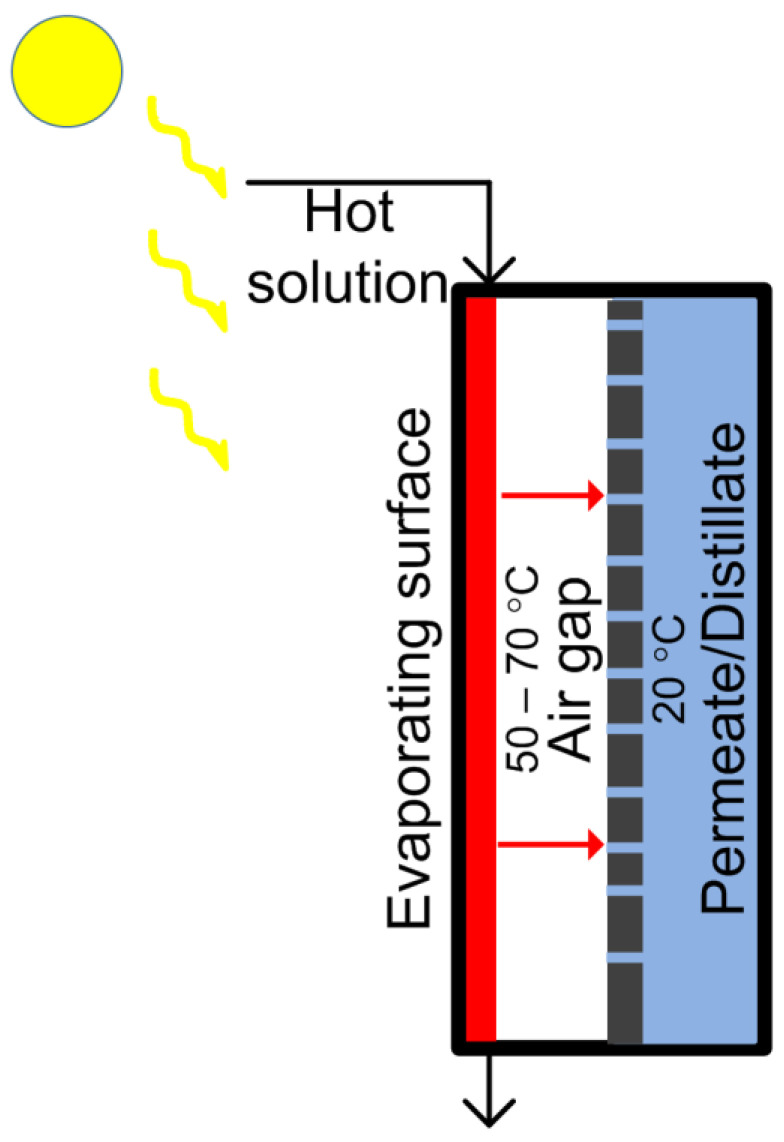
Film distillation with a porous (membrane) condensing surface (PMC). The initial solution is heated in an external heat exchanger and fed hot into the separation module [18].

A feature of the installation is the use of film distillation with a porous condensing surface (FD-PC) (Figure 1). The evaporating surface is used, directly heating the solution supplied at ambient temperature. A thin liquid film was formed by supplying a saline solution through a splitter in the upper part of the module to the evaporating surface. The solution spread as a result of the liquid wetting of the evaporating surface and formed a thin liquid film flowing down the module under the action of gravity. The water evaporated from the film surface was condensed on the porous membrane and discharged into the coolant chamber under the action of transmembrane hydrostatic pressure.

**Figure 2:**
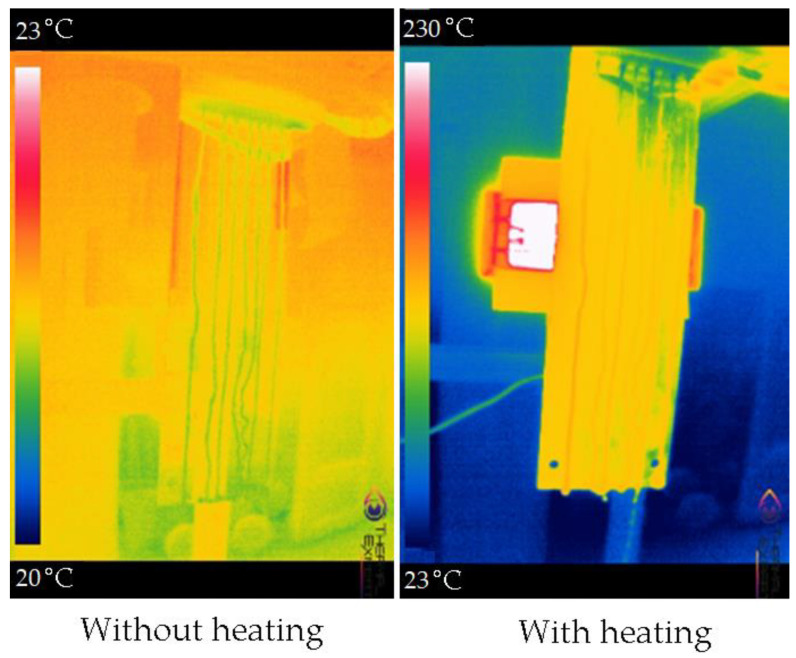
Thermal photos of the evaporating surface when water is supplied without heating and with heating of the surface.

At the same time, when choosing the material of the evaporating surface, it is especially important to take into account the resistance to the chemical effects of saturated brines, to heating (and, in general, temperature differences), and other physicochemical as well as biological influences of the work and environment. SS304 stainless steel was chosen as a similar material. However, the test run revealed too small a spreading area of the water flow over the evaporating surface (Figure 2). The test run consisted of pumping distilled water through the system in conditions without a heating lamp and with a lamp. As can be seen from Figure 2, about 10% of the evaporation surface area is filled with a salt solution.

**Figure 3:**
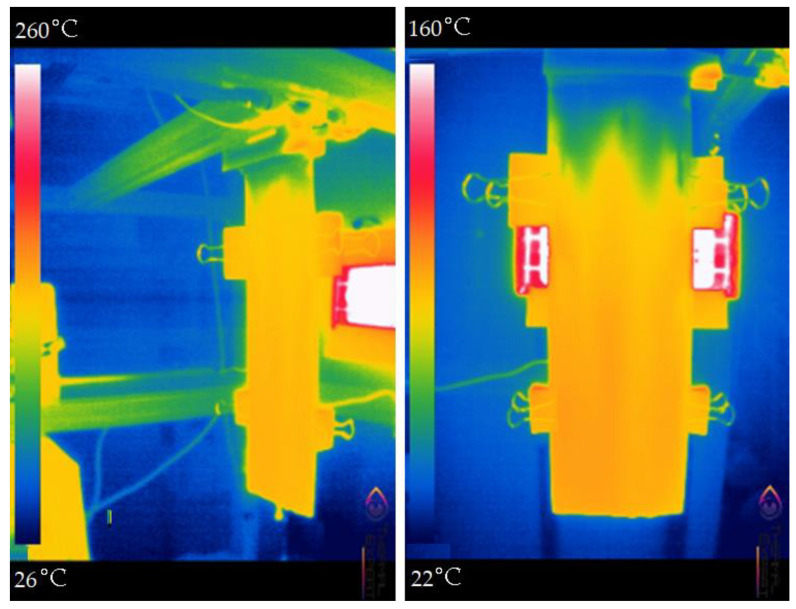
The surface area of liquid spreading over plastic with a polymer mesh flow divider. It can be seen that, in this configuration, almost the entire evaporating surface will be covered with the treated liquid.

Taking into account the previous experience in the development of FD-PC modules [11,23], a combined surface consisting of a base made of polymer material PETG with a soldered polyethylene mesh that ensures the spreading of the treated solution over the evaporation surface was used. As a result (Figure 3), it was possible to obtain an evaporating surface having the qualities of a solid plate, but with almost 100% of the surface area of the liquid spreading; the parameters of the selected mesh were as follows: thickness 0.6 mm, cells 0.3 × 0.3 mm, and irrigation area: ~100%. 

### 2.4. Installation of Film Distillation with a Porous Condensing Surface

The laboratory installation of film distillation with a porous condensing surface, used for concentrating aqueous solutions of salts, was previously described in detail in the patent [24] and the work of Golubev et al. [18,24].

**Figure 4:**
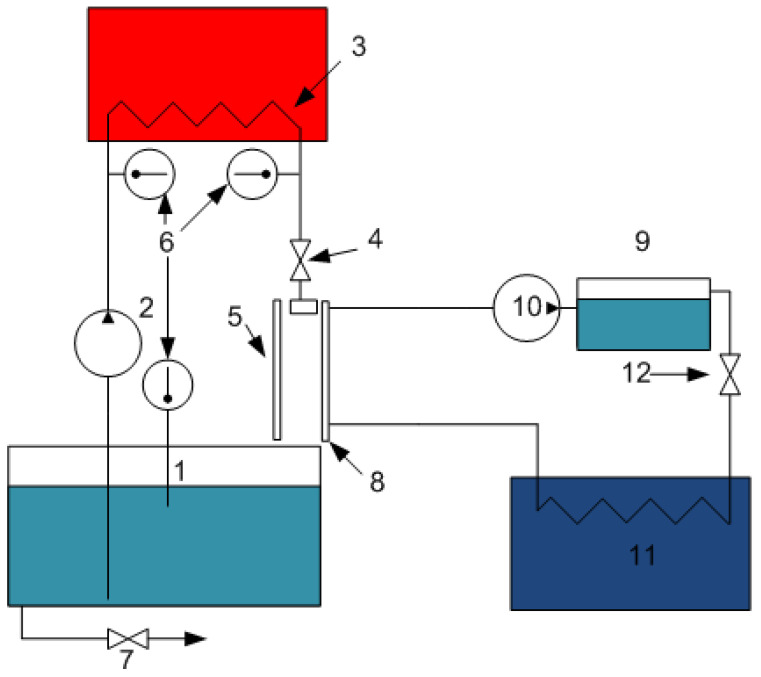
FD-PC installation diagram. 1—tank with treated liquid (seawater), 2—pump, 3—coil, 4—control tap, 5—evaporating surface, 6—thermometers, 7—drain tap, 8—porous condenser, 9—container with distilled water, 10—pump, 11—chiller, 12—control tap.

The principle of operation of the FD-PC system is described below (Figure 4). From tank 1, the treated liquid (seawater) is supplied to the evaporating surface 5 by pump 2 through the coil of the heating element (thermostat) 3. Flow control is carried out by means of a tap 4. The heating temperature of the treated solution (seawater) varies from 40 to 80 °C. Temperature control is carried out using thermometers 6. The solution was drained after the experiment through a tap 7. Water vapor condenses on a porous surface 8. At the same time, a stream of coolant/permeate is constantly moving from a container with distilled water 9 through a tap 12 and a chiller 11 with a pump 10.

The feed solution was circulated in the module at a temperature of 50 to 70 °C. The coolant was circulated at a temperature of 20 °C with a constant flow rate of 0.3 L/min through a module with a membrane (porous) condenser. The evaporative surface is a steel plate with an area of 210 cm^2^, along which a saline solution flowed. The width of the air gap was 3 mm.

An aqueous solution of sodium chloride with an initial concentration of 35 g/kg was used as a model solution, which corresponds to the salinity of seawater off the coast of Vietnam. Distilled water was used to prepare the solutions. The evaluation of the working area of the evaporating surface was carried out from photographs obtained using a thermal imager (Thermal Expert TE-Q1). Using the Gwyddion software (ver. 2.62, Czech Metrology Institute, Brno, Czech Republic) [25], the calculation of the surface area filled with a hot solution was performed.

In addition to the elements directly described above, there are a large number of other construction elements in the system, in particular pipelines, fittings, and cranes. Checking the stability of these construction elements in the climatic conditions of Vietnam was also part of the presented work.

Separately, it is worth focusing on the material of the porous condenser. Previously, sintered stainless steel plates were used as a porous condensing surface in modules of this design. However, in the previous stage of the work, it was found that this material can be unstable in the tropical climate of Vietnam.

### 2.5. Biocide Additives

The polymer films with biocide additives were fabricated by introduction of 0.5 wt.% of PGMGH-MMT (by polymer weight) in casting solution (20 wt.% for polysulfone, 20 wt.% polyestersulfone, or 25 wt.% for polyphenylene sulfone in NMP). The resulting mixture was stirred for 3 days (until the polymer dissolved). The films were cast on glass and leveled with a squeegee. The obtained samples were dried in a drying cabinet at 80 °C for one day. The thickness of the resulting films was 50–100 micron. Porous membranes formed using the phase inversion method by releasing a film of solution on glass into a precipitation bath with water.

### 2.6. Research Methods

The pore size distribution (PSD) was measured by a liquid–liquid displacement porosimetry (LLDP) [26] using porometer POROLIQ 1000 ML (Porometer, Berlin, Germany).

Scanning electron microscopy (SEM) was used to characterize the structure and morphology of the polymer blend membranes. SEM was carried out on a Thermo Fisher Phenom XL G2 Desktop SEM (Waltham, MA USA). Cross sections of the membranes were obtained in liquid nitrogen after preliminary impregnation of the specimens in isopropanol. A thin (5–10 nm) gold layer was deposited on the prepared samples in a vacuum chamber (≈0.01 mbar) using a desktop magnetron sputter, the Cressington 108 Auto Sputter Coater (Cressington Scientific Instruments, Inc., Cranberry Twp, PA, USA). The accelerating voltage during image acquisition was 15 kV.

The composition of the solution was determined by the electrical conductivity, which was measured using a portable conductometer WTW Cond 3210 (WTW, Weilheim, Germany) with a WTW TetraCon 325 cell (WTW, Weilheim, Germany) (the range of electrical conductivity measurement zones is 1 mcm/cm–2 cm/cm, accuracy ±0.2%) according to the pre-constructed calibration curves.

### 2.7. Simulation in Simulink MatLab Program

**Figure 5:**
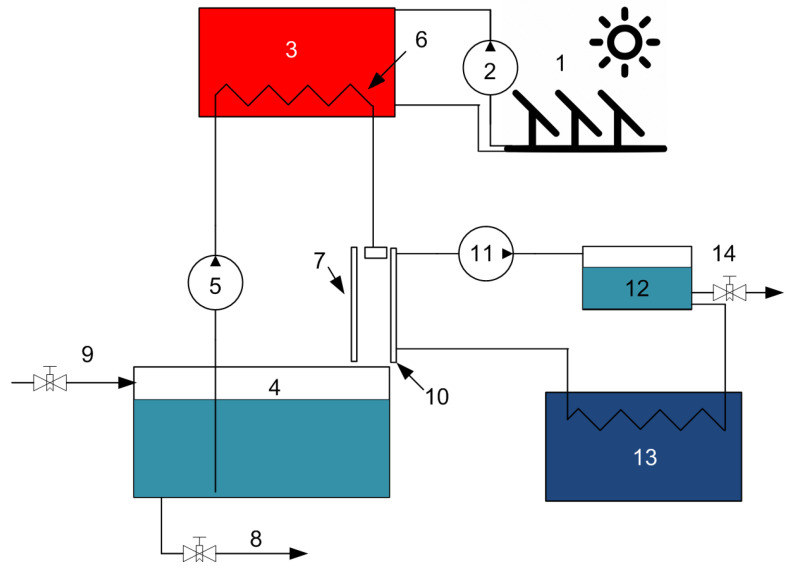
Diagram of the simulated FD-PC system. 1—solar collectors, 2—pump for pumping heat-carrying fluid, 3—heat accumulator tank, 4—desalinated water tank, 5—seawater supply pump, 6—heat exchanger, 7—evaporating surface (FD), 8—removal of excess concentrate, 9—power supply (seawater), 10—porous condensing surface (PCS), 11—coolant/permeate pumping pump, 12—coolant/permeate tank, 13—chiller, 14—clean water outlet.

The principle of operation of the FD-PC installation model was described in detail in previous articles by a team of authors on the subject of membrane and film distillation [11,17,27]. Below (Figure 5) is a schematic diagram of the FD-PC installation model. The system consists of two subsystems: heat storage and working. In the first subsystem, with the help of solar collectors (panels with an area of 2 m^2^), the heat accumulator (10,000 kg tank) is heated with clean water. The total heat carrier flow was equal to the sum of the flows of all collectors (flow for one collector: 50 kg of coolant per hour). The working solution is heated in a heat exchanger and fed into a system of evaporative thin-film modules (the total area of the modules varied from 1 to 25 m^2^), in which a part of the solvent (water) is released through the coolant circuit. The remaining concentrated solution is returned to the container with the separated mixture (the working mass of the solution is 50 kg). To remove excess saline solution of a high concentration in the system, a relief valve was provided, which opened when a concentration close to saturation (24–26 wt.%) was reached in the system. The solar collector system was turned off at night but, because of the presence of heat storage of a sufficiently large volume, the film distillation module worked around the clock; at night, the distillate evaporation flows decreased. 

As part of this work, a computational analysis of the system’s performance was also carried out. In particular, the calculation of the most efficient arrangement of evaporative modules was carried out. In previous works, systems consisting of single-stage evaporative modules were considered. Thus, it was assumed that, after passing the evaporator, the solution immediately returned to the tank with the treated solution. Within the framework of this work, it was assumed that similar performance can be achieved using a multi-stage installation, where the solution coming out of the higher stage can become a feeder for the lower one.

## 3. Results and Discussion

### 3.1. Testing of Samples and Construction Elements for Stability in the Tropical Climate of Vietnam

**Figure 6:**
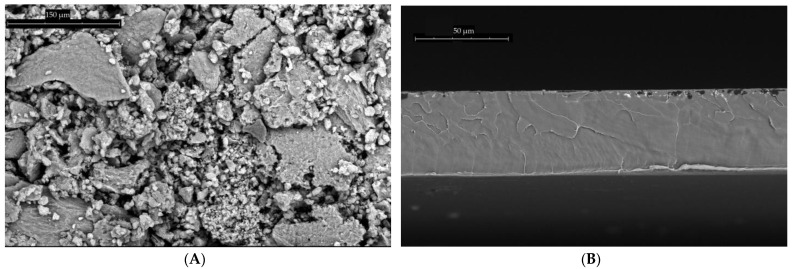
(**A**) SEM of the PHMGH-MMT biocide; (**B**) the cross section of the PS film with the addition of 0.5% PHMGH-MMT.

For some of the samples presented, in particular ABS, Polyamide-6, and Stainless steel SS305, the pipeline elements and fittings were studied earlier, and the results of the study were presented in [11]. A polyelectrolyte material, polyhexamethylene guanidine hydrochloride, used in this study was synthesized at TIPS RAS in accordance to the method described in [28], deposited on the layered clay mineral montmorillonite (abbreviated PHMGH-MMT). The content of polyhexamethylene guanidine hydrochloride in the biocide was 30 wt.%. Figure 6 shows micrographs of the surface and cross sections of membranes containing biocidal additives. Figure 6B,C clearly demonstrate the change in the membrane structure with the addition of more additives. In December 2021, the polymer film and membranes made of polysulfone, polyestersulfone, and polyphenylene sulfone with and without addition of biocide were exposed to the environment in Vietnam. The results of sample exposure are presented in Table 1.

Thus, as a result of this study, materials suitable for an FDPC installation in the conditions of the tropical climate of Vietnam were designed.

### 3.2. The Process of Film Distillation with a Membrane-Condensing Surface

To evaluate the efficiency of the FD-PCS module, an experiment was conducted on the concentration of NaCl salt solution at the installation described above. The experiment was carried out for two different heating temperatures of the solution before being applied to the evaporating surface: 55 and 65°. At the same time, the temperature in the coolant/permeate circuit remained the same at 20 degrees. Figure S2 in Supplementary Materials shows one of the key characterizing parameters of the FD-PC system—the performance of the membrane module, expressed in kg of evaporated liquid per m^2^ of evaporation area per hour.

One of the most important goals of this work was to determine the effective modes of operation of the FD-PC system in a tropical climate in comparison with similar experiments conducted in the Russian middle zone, as well as to check the stability of FD-PC installations for transportation, the principal possibility of using installations of this configuration outside the working laboratory. To evaluate the effectiveness of the FD-PC module, an experiment was conducted on the concentration of NaCl salt solution directly in the climate of Vietnam. The results of the experiment are presented in Table 2. One of the key characterizing parameters of the FD-PC system is described—water flux, expressed in kg of evaporated liquid per m^2^ of evaporation area per hour. The feed solution was circulated in the module at a temperature near of 60 °C. The coolant was circulated at a temperature near of 20 °C with a constant flow rate of 0.36 L/min through a module with a membrane (porous) condenser. The evaporative surface is a plastic plate made of PETG with an area of 196 cm^2^, along which a saline solution flowed. The width of the air gap was 3 mm.

An aqueous solution of sodium chloride with an initial concentration of 35 g/kg was used as a model solution, which corresponds to the salinity of seawater near the coast of Vietnam. 

**Figure 7:**
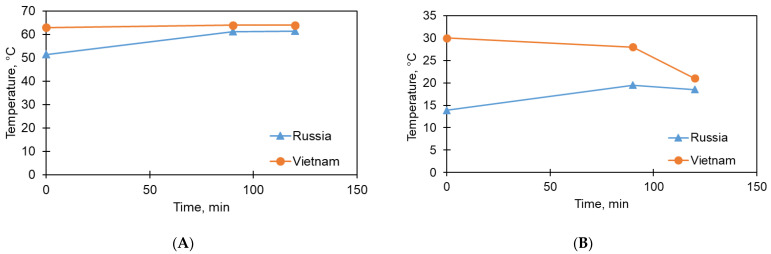
Dependence of the heating temperature of the solution (**A**) and the cooling temperature of the coolant/permeate (**B**) for experiments in Russia (triangles) and Vietnam (circles).

The results obtained show good repeatability along the evaporation flow and permeate absorption flux. It is important to take into account the thermal stability of the operating mode of the installation. Figure 7 presents experimentally determined values of the heating temperature of the solution (Figure 7A) and the cooling temperature of the coolant/permeate (Figure 7B). The maximum error of temperature measurement is 0.5 °C. The time measurement margin of error was no more than 1 min.

It is clearly noticeable that, at the same time, because of the climatic features of Vietnam, the heating temperature of the solution remains more stable. At the same time, the differences in temperature remain quite low. In contrast to heating, permeate cooling in a tropical climate is more complicated, which is expressed in the gradual cooling of clean water in the coolant circuit from 30 °C to 20 °C. A slower output to operating temperatures in this case is one of the most likely reasons for the appearance of deviations in the permeate flux in favor of higher indicators in studies conducted in Russia (despite the fact that evaporation flows differ slightly).

These results demonstrate good reproducibility of experiments; however, a number of features affecting the operating mode of the system were also identified.

### 3.3. Modeling of Desalination System

**Figure 8:**
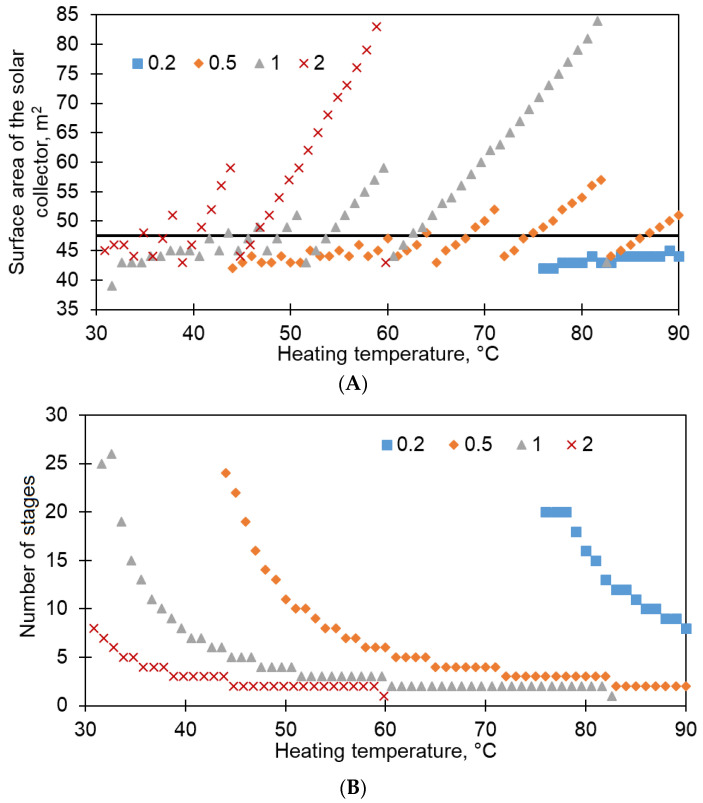
The dependence of the surface area of the solar collectors (**A**) and the number of stages of evaporation modules (**B**) depending on the heating temperature of the solution required to ensure a capacity of at least 12 L/m^2^h for various variants of the evaporator surface area (as well as the area of the condenser). The dotted line marks the average median of the surface area of the solar collector.

The calculation of the optimal number of stages allows, on the one hand, to minimize the heating temperature of the solution and, on the other hand, to ensure high system performance. The low heating temperature of the solution is advisable both from the point of view of heat preservation in the heat accumulator tank and from the point of view of increasing the efficiency of solar collectors [29]; thus, the highest efficiency is observed with a smaller difference in the temperature of the coolant in the collector and the ambient temperature. However, as shown in [18], the performance of the FD-PC system drops significantly when the heating temperature of the solution decreases. Another important parameter is the power of the mass flow, for which it is necessary to use electric pumps. It is obvious that several stages standing sequentially under each other will require the same mass flow. To determine the optimal configuration, the simulation of the performance of FD-PC systems for various variants of the evaporation surface area was carried out. At the same time, the production parameter remained unchanged during modeling: at least 12 L/m^2^h. The choice of productivity is based on the evaluation of the performance of other desalination methods, in particular reverse osmosis [6]. The simulation result is shown in Figure 8.

According to the results, there are such configurations of evaporative modules that allow, while maintaining the system performance, to maintain low heating temperatures of both the solution itself and the heat accumulator tank. At the same time, with an increase in the surface area of the evaporation stage, the required number of stages obviously decreases, which will avoid the construction of bulky structures.

Moreover, as noted earlier, FD-PC systems in the presence of collector systems require energy only to power pumps. In this case, three pump blocks are needed to ensure the operation of the system:

(1) providing pumping of water in solar collectors;
(2) providing pumping of the working solution;
(3) providing coolant/permeate pumping.

Thus, it is possible to carry out a simple calculation of the energy consumption required for the operation of the system. The flow data for the evaporation modules are given above. Data on the power flows of solar collectors are given in an earlier work [11]. In Table 3, the values of specific energy consumption for different systems are presented for comparison (FD-PC—film distillation with a porous condenser, ED—electrodialysis, RO—reverse osmosis, FO—forward osmosis). To estimate the energy costs, it was assumed that the heating of the liquid was carried out exclusively by membrane collectors. Thus, third-party energy sources were required only to ensure the operation of the pumps. At the same time, in order to provide series-connected modules, a pump with a performance similar to that of a pump is required to ensure the operation of a single module. Thus, taking into account the experimental values of the flows indicated above, the flows were calculated. In accordance with this, the capacity of a commercial pump, similar in performance, was selected. The power of pumps providing power to solar collectors with coolant was also determined. Finally, based on experimental data, the pump capacity in the coolant circuit was determined. All pumps used in this installation are necessary only to ensure a given flow, but do not require the creation of increased pressure. Pumps are also resistant to seawater.

It is worth noting that the efficiency of solar collectors depends on the intensity of solar radiation. At lower radiation rates, with a similar number of collectors, a smaller amount of clean water can be produced and, consequently, the SEC will be higher. However, the SEC value for FD-PC will still remain below the analogs. This allows us to talk about the potential efficiency of FD-PC systems, especially in conditions of a lack of electricity. Thus, based on the results of the simulation, more efficient plant configurations were determined.

## 4. Conclusions

The stability of a number of materials was analysed in relation to the conditions of the tropical climate of Vietnam. In particular, the stability of membranes made of polysulfone, polyethersulfone, and polyphenylenesulfone with the addition of biocide PGMGH-MMT was analyzed. As a result of a long (3 months) exposure of the samples, it was found that samples with a concentration of biocide of 0.5 wt.% possessed lower biofouling, in contrast to the neat membranes. In this regard, the porous condenser based on polysulfone with the addition of a biocide active substance can be considered as a perspective alternative to the metal porous flat-sheet membranes used previously in our study. The experiments showed high reproducibility of the results; in particular, the evaporation rate was (4.9/3.0) kg/m^2^h in Russia and (4.1/2.0) kg/m^2^h in Vietnam. However, the design of the condensation compartment needs further optimisation to reduce the effect of temperature fluctuation on its performance. Using experimental data, a simulation of the operation of the integrated FD-PC system using solar collectors as the main heating elements was carried out. Calculations have shown high energy efficiency of the system. Therefore, the specific energy costs are 0.1–0.5 kWh/m^3^. Such low energy costs allow us to talk about the competitiveness of desalination plants based on FD-PC. At the same time, reverse osmosis or electrodialysis plants are more productive, but also significantly more energy-intensive. 

In the future, as part of this work, it is planned to improve the condenser cycle to increase system performance and thermal stability. Of particular interest is the active use of biocide-containing polymers as a porous condenser.

## Figures and Tables

**Table 1:** Long-term stability of materials in a tropical climate: “+” the sample remained intact; “+/−” the sample is partially deformed; “−” the sample is destroyed. Exposure time—3 months.

Sample	Dam Bai		Hoa Lak		Con Zo	
	Sun	Sea	Shadow	Ground	Sun	Shadow
Polysulfone	+	+/−	+	+/−	+	+
Polyethersulfone	+	+/−	+	+/−	+	+
Polyphenylene Sulfone	+	+/−	+	+/−	+	+

**Table 2:** The performance of FD-PC modules depends on the conditions of the experiment. Heating temperature −60 °C, permeate temperature −20 °C, permeate flow −0.36 kg/min, and flow of the treated solution −0.17 kg/min.

Location of the Experiment	Evaporation Flow, kg/m^2^h	Permeate Flux, kg/m^2^h
Russia	4.9	3.0
Vietnam	4.1	2.0

**Table 3:** Comparison of specific energy consumption for common methods of water desalination.

Method	Specific Energy Consumption, kWh/m^3^	Source
FD-PC	0.1	This work
ED	2.64	[8]
RO	2.23	[6]
FO	0.24	[30]

## Data Availability

The data presented in this study are available upon request from the corresponding author.

## Supplementary Materials

The following supporting information can be downloaded at: https://www.mdpi.com/article/10.3390/membranes13020163/s1. Detailed data relating to the study is presented in Supplementary Figure S1 and Figure S2.
