# Supplementary material for: Film Distillation with a Porous Condenser for Seawater Desalination: Evaluation of Materials’ Stability in the Tropical Climate of Vietnam
Source: Membranes (Basel). 2023 Jan 27;13(2):163. doi: 10.3390/membranes13020163 (PMC9966059; doi:10.3390/membranes13020163)
Supplement: Supplementary file 1 [file membranes-13-00163-s001.zip › membranes-2132813-supplementary.pdf]

Photos of samples (Figure S1) exposed to seawater show biofouling of samples without biocide (Figure S1A) and visually observed less fouling of samples with 0.5 biocide additives (Figure S1B). As can be seen, the film with biocide remains smooth and characterized by less fouling.

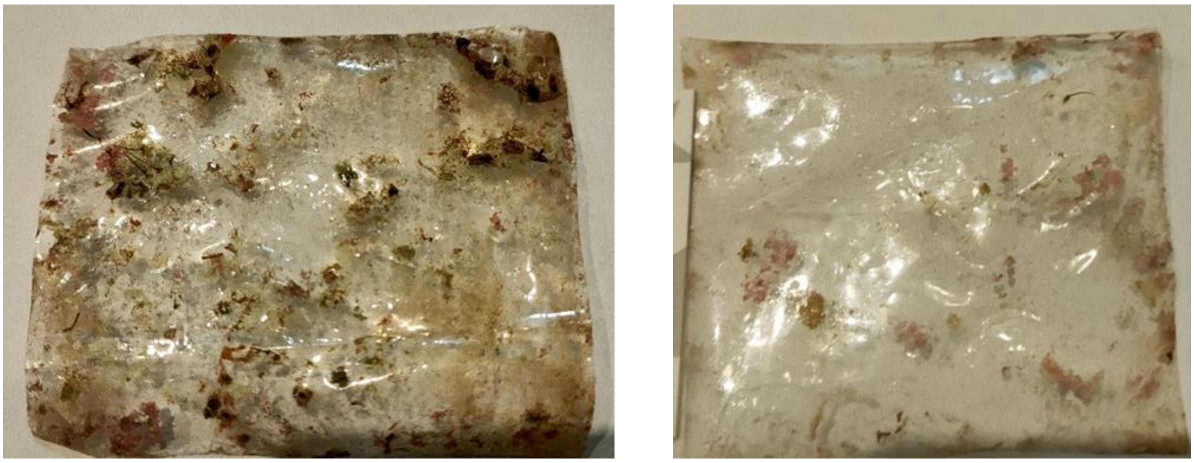

A	B

Figure S1. Samples of polysulfone films with the addition of various concentrations of biocide, were exposed in Dam Bai, in the sea, for 3 months. A) polysulfone 20% - 0% biocide, B) polysulfone 20% - 0.5% biocide.

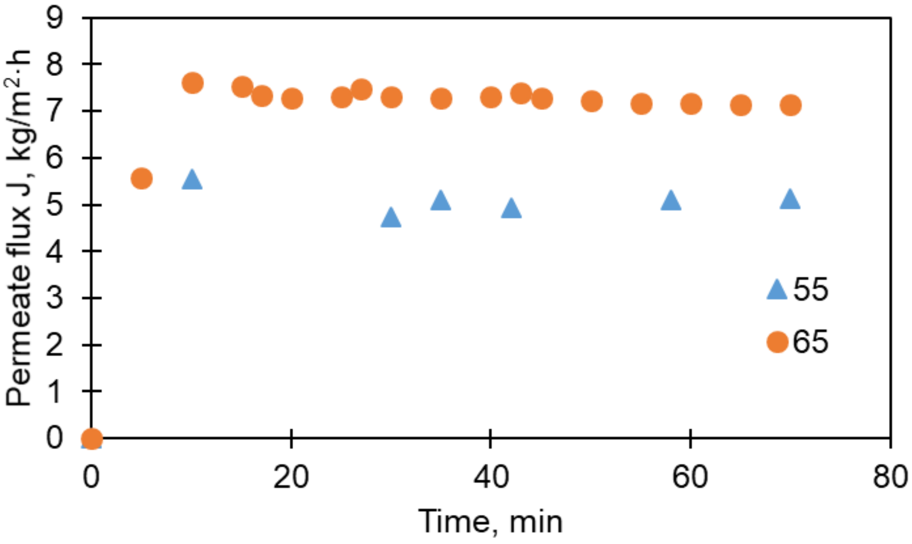

Figure S2. Experimental data on the performance of the FD-PC module depending on the time of the experiment for two solution heating temperatures: 55 (triangles) and 65 (circles) degrees Celsius.
